# 

**Published:** 1896-07-18

**Authors:** 


ABSOLUTELY PURE, therefore BEST,

CADBURY'S COCOA
"The standard of highest purity at present attainable."—*Lancet.*

CADBURY'S COCOA

NO ALKALIES USED.

### THE HOSPITAL

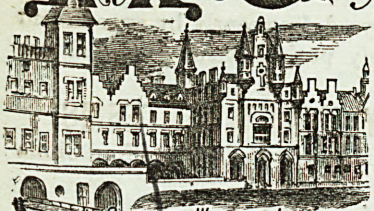

GLASGOW WESTERN INFIRMARY

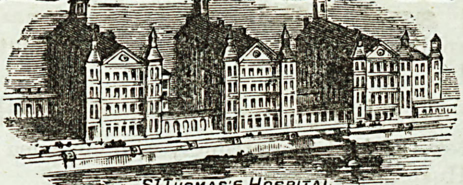

ST THOMAS'S HOSPITAL

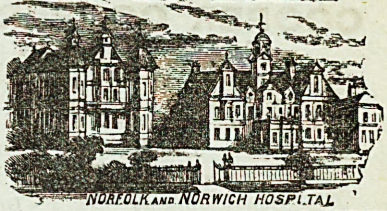

NORFOLK AND NORWICH HOSPITAL

No. 512. Vol. XX.

Saturday, July 18th, 1896.

WITH EXTRA NURSING SUPPLEMENT.

[Registered at the General Post Office as a Newspaper for transmission in the United Kingdom and Abroad.]

PRICE TWOPENCE

Per Annum, 10s. 6d., post free.

Monthly Parts, 6d.; post free, 8d.

